# Diagnosis of left ventricular hypertrophy using non-ECG-gated ^15^O-water PET

**DOI:** 10.1007/s12350-021-02734-3

**Published:** 2021-07-20

**Authors:** Jens Sörensen, Jonny Nordström, Tomasz Baron, Stellan Mörner, Sven-Olof Granstam, Mark Lubberink, Lars Tolbod, Jeffrey van den Berg, Frank A. Flachskampf, Tanja Kero, Peter Magnusson, Hendrik J. Harms

**Affiliations:** 1 Department of Surgical Sciences, Radiology & Nuclear Medicine, Uppsala University, Uppsala, Sweden; 2 Centre for Research and Development, Region Gävleborg/Uppsala University, Gävle, Sweden; 3 Department of Medical Sciences, Clinical Physiology and Cardiology, Uppsala University, Uppsala, Sweden; 4 Nuclear Medicine and PET, Institute of Clinical Medicine, Aarhus University, Aarhus, Denmark; 5 Department of Public Health and Clinical Medicine, Umeå University, Umeå, Sweden; 6 Cardiology Research Unit, Department of Medicine, Karolinska Institutet, Stockholm, Sweden; 7 PET Center, Entrance 86, Uppsala University Hospital, 751 85 Uppsala, Sweden

**Keywords:** Cardiac remodeling, Left ventricular hypertrophy, ^15^O-water, Positron emission tomography, Wall thickness

## Abstract

**Aim:**

To develop a method for diagnosing left ventricular (LV) hypertrophy from cardiac perfusion ^15^O-water positron emission tomography (PET).

**Methods:**

We retrospectively pooled data from 139 subjects in four research cohorts. LV remodeling patterns ranged from normal to severe eccentric and concentric hypertrophy. ^15^O-water PET scans (*n* = 197) were performed with three different PET devices. A low-end scanner (66 scans) was used for method development, and remaining scans with newer devices for a blinded evaluation. Dynamic data were converted into parametric images of perfusable tissue fraction for semi-automatic delineation of the LV wall and calculation of LV mass (LVM) and septal wall thickness (WT). LVM and WT from PET were compared to cardiac magnetic resonance (CMR, *n* = 47) and WT to 2D-echocardiography (2DE, *n* = 36). PET accuracy was tested using linear regression, Bland–Altman plots, and ROC curves. Observer reproducibility were evaluated using intraclass correlation coefficients.

**Results:**

High correlations were found in the blinded analyses (*r* ≥ 0.87, *P* < 0.0001 for all). AUC for detecting increased LVM and WT (> 12 mm and > 15 mm) was ≥ 0.95 (*P* < 0.0001 for all). Reproducibility was excellent (ICC ≥ 0.93, *P* < 0.0001).

**Conclusion:**

^15^O-water PET might detect LV hypertrophy with high accuracy and precision.

**Supplementary Information:**

The online version contains supplementary material available at 10.1007/s12350-021-02734-3.

## Introduction

Left ventricular (LV) hypertrophy is a strong predictor of future cardiovascular risk, as shown in the Framingham and MESA cohort studies.^1^,^2^ In a recent 15-year follow-up in the MESA trial, increased LV mass (LVM) was strongly associated with long-term development of heart failure, myocardial infarction and cardiovascular death, independently of traditional risk factors, and significantly better than calcium scoring by CT.^3^ Early detection of LV hypertrophy therefore provides an opportunity for risk reduction.^4^ A first diagnosis of LV hypertrophy is typically established using 2D-echocardiography (2DE), but mainly by measuring septal wall thickness (WT), and there are known issues with the accuracy and reproducibility of LVM measurements using 2DE.^5^ However, a finding of isolated increased WT by 2DE should trigger further work-up to rule out a diagnosis of primary cardiomyopathy (e.g., cardiac amyloidosis (CA) and hypertrophic cardiomyopathy (HCM)) even when LVM is not increased, which makes WT an important parameter in its own right. Cardiac magnetic resonance imaging (CMR) is the acknowledged gold standard for assessment of LVM and WT.

Left ventricular hypertrophy is strongly linked to abnormal myocardial perfusion,^6^,^7^ which makes combined evaluation of quantitative myocardial perfusion and LV hypertrophy by positron emission tomography (PET) a clinically appealing approach. Worldwide, the most commonly used tracer for cardiac PET is ^82^Rb-rubidium (82Rb), which was recently shown to produce reliable estimates of LVM with dedicated post-processing software^8^ with a high degree of reproducibility.^9^,^10^

^15^O-water PET is considered the gold standard for absolute quantification of regional MBF and has high accuracy in detecting hemodynamically significant coronary artery disease (CAD).^11^-^13^^15^O-water is approved for clinical use in some countries in Europe and Asia, and our site routinely perform cardiac ^15^O-water PET for CAD evaluation since 2012, However, there is currently no method by which LVM and WT can be measured from a ^15^O-water scan.

Contrary to 82Rb and all other radiopharmaceuticals used for cardiac perfusion imaging, ^15^O-water is freely diffusible and rapidly equilibrates with the large endogenous water pool. Consequently, static images of ^15^O-water obtained more than 1 minute after injection hold no contrast. Kinetic analysis of dynamic ^15^O-water data are mandatory and results in robust formation of 3D parametric MBF images suitable for clinical use.^14^ ECG-gated first-pass blood pool images were shown to provide an accurate workaround for measurements of LV volumes and ejection fraction.^15^,^16^ First-pass images cannot, however, be used to assess LV mass.

**Figure 1:**
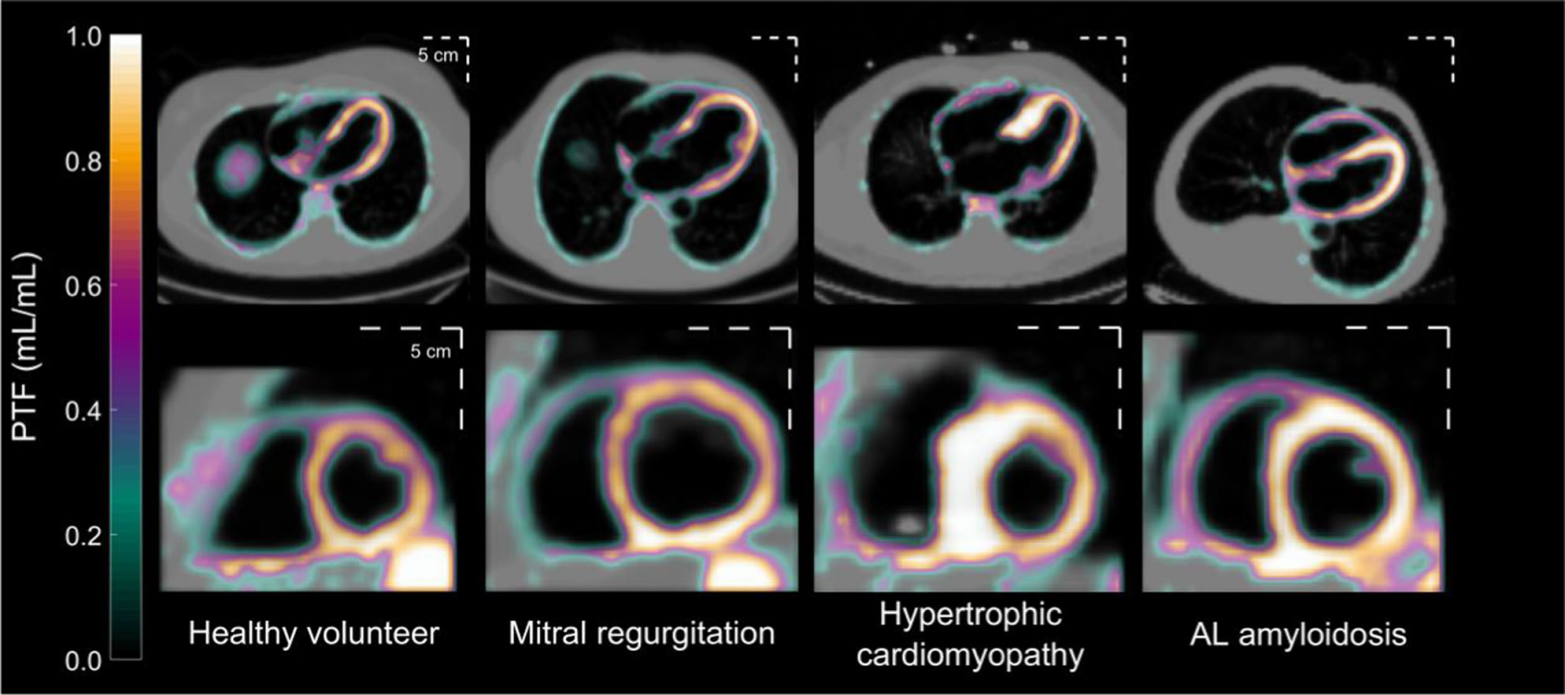
Parametric images of Perfusable Tissue Fraction (PTF, calculated from ^15^O-water PET) from study subjects with different patterns of left ventricular remodeling. Upper row: PTF fused with anatomical tissue fraction, in which blood volume was subtracted from normalized computer tomography. Lower row: short-axis mid-ventricular PTF. All panels share the same color scale. A PTF value of 1 mL/mL indicates that a voxel contains 100% perfused tissue throughout the cardiac cycle. A metric scale is inserted

**Figure 2:**
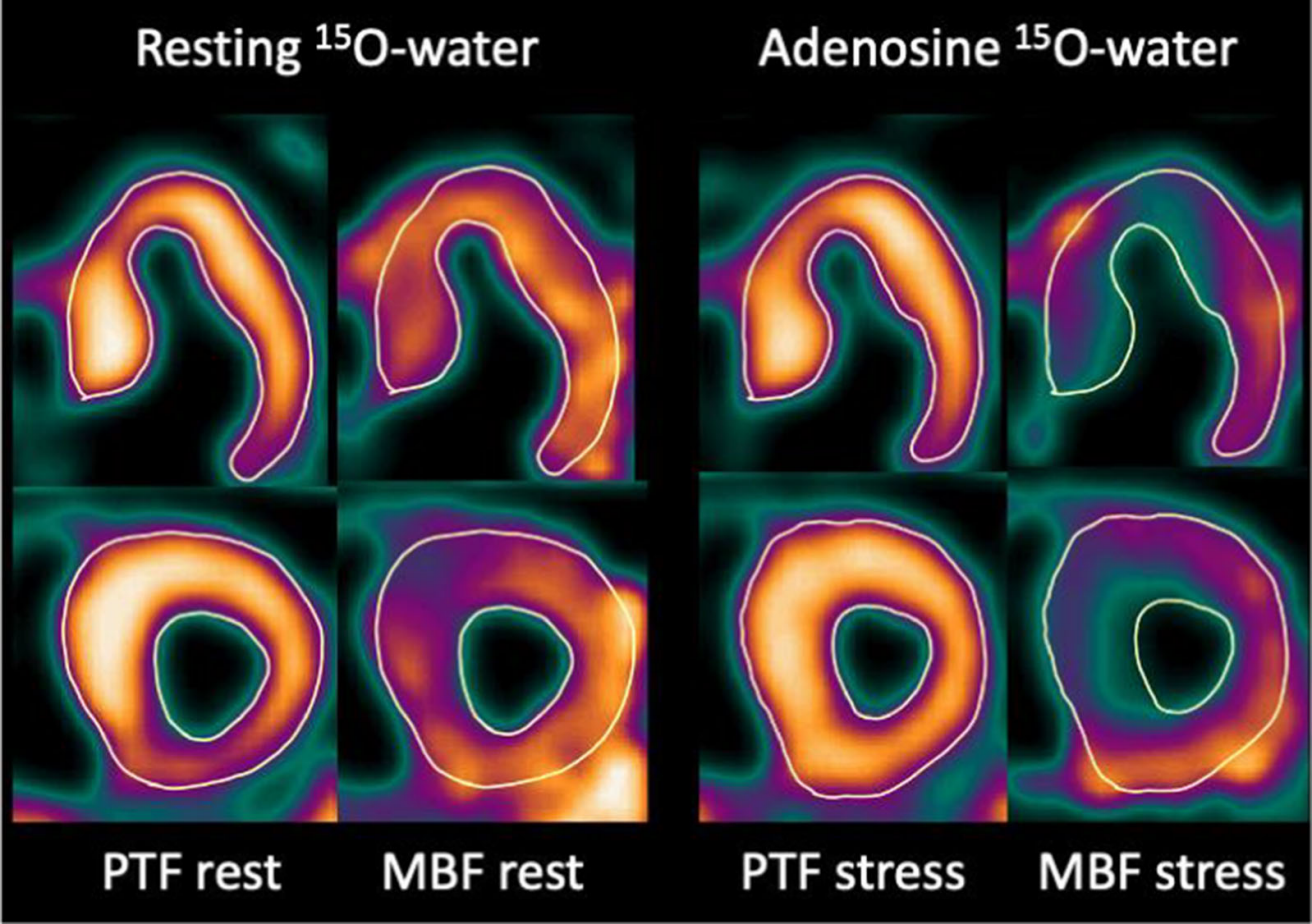
Example parametric ^15^O-water PET images of Perfusable Tissue Fraction (PTF) and myocardial blood flow (MBF) in a subject with hypertrophic obstructive cardiomyopathy, scanned at rest and during adenosine infusion stress. Rest MBF shows a mild perfusion defect in the anterior interventricular septum and stress MBF severe subendocardial perfusion deficits in septal and apical regions. PTF images at rest and stress are similar. Septal wall thickness by 27 mm PET and 25 mm by 2D-echocardiography

The pharmacokinetic model used to generate parametric MBF images also results in parametric images of the perfusable tissue fraction (PTF), a correction for the partial volume effect (reduced signal recovery) induced by thin walls, wall motion and scarring.^17^,^18^ As shown in Fig. 1, PTF images depict the average position of the heart and provide high-contrast structural information related to cardiac configuration. Such images are useful for myocardial delineation in a consistent manner, which is required for MBF measurements. In clinical practice, PTF images for wall delineations enhance the interpretation of MBF images in cases with highly irregular regional distribution of perfusion (see Fig. 2). In recent work, parametric images derived from PET using ^11^C-acetate, a tracer with high myocardial retention during the first few minutes after delivery, were shown to produce highly accurate estimates of LVM and WT without ECG-gating, compared to CMR.^19^ A similar approach may enable LVM and WT estimates with ^15^O-water PET when parametric PTF images are used.

The aim of this study was to investigate the feasibility of measuring LVM and WT by delineating the LV wall on non-gated parametric PTF images, and to study the impact of different cardiac remodeling patterns and scanners on the validity of these measurements.

## Methods

### Subjects

**Figure 3:**
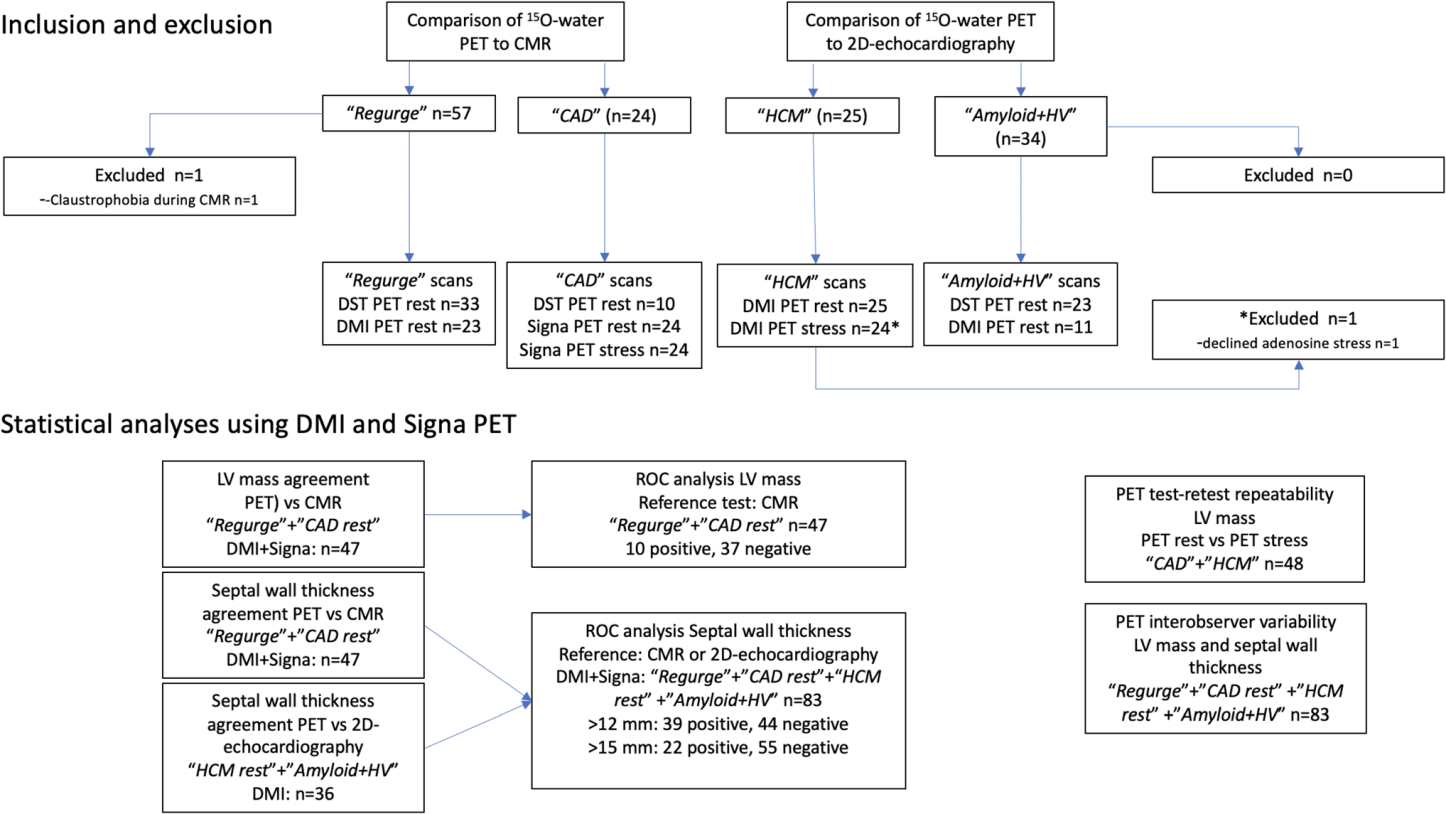
Modified STARD diagram detailing included groups with reasons for excluding subjects from further analysis and planned statistical analyses. CMR: cardiac magnetic resonance

Subjects were recruited from four different observational research studies, all involving secondary end-points associated with developing ^15^O-water PET methodology for diagnosing structural heart disease. The studies were performed in one center during 2015 to 2018. Eligible subjects were defined by either a planned near-simultaneous cardiac magnetic resonance imaging (CMR-comparison) or an echocardiography (echo-comparison) as part of the study. CMR is the gold standard for cardiac geometry. We included the echo-comparison to study the PET approach in subjects with known concentric and irregular hypertrophy due to primary cardiomyopathies, such data were not available for comparison to a same-day CMR. In total, 140 subjects were screened for inclusion/exclusion criteria and data from 139 subjects were eventually used in the current study. A modified STARD diagram with study groups and planned statistical analyses is shown in Fig. 3. Method development was performed using scans obtained with an older device (GE Discovery ST16, DST), which was installed 2003 and discarded early 2017. When we started to analyze data obtained with more recent PET devices, we decided to perform separate statistical analyses for each of the three scanners used, with a primary focus on results from the contemporary scanners with blinded observers. Results from analyses using the DST scanner were summarized and discussed in the main text with further details in supplementary material. Patient characteristics are provided in Table 1.

**Table 1:** Subject grouping characteristics

Cohort	*N*	Male/female	Age (years)	HR	BSA	PET scanner	
				(min^−1^)	(m^2^)	DST (training)	DMI + Signa (blinded)
CAD	24	16/8	64 ± 9	67 ± 7	1.9 ± 0.2	10*	24
Regurge	56	50/6	64 ± 9	62 ± 9	2.0 ± 0.2	33	23
Amyloid+HC	34	23/11	65 ± 10	65 ± 13	1.9 ± 0.2	23	11
HCM	25	19/6	56 ± 13	60 ± 8	2.1 ± 0.3	0	25

*BSA*, body surface area; *HR*, heart rate

*10 subjects were scanned both with DST and Signa

The following subject groups were included in the blinded analysis:

- LVM and WT from PET were compared to the gold standard of CMR in a cohort consisting of 47 subjects, in which PET and CMR were performed either simultaneously with a GE Signa PET/MR (Signa) or on the same-day using a GE Discovery MI PET/CT (DMI) and standalone CMR.
  1. The PET/MR cohort consisted of 24 subjects with suspected or known coronary artery disease (“CAD” in Table 1 and Fig. 3). These subjects underwent both rest and adenosine-induced stress scans in one session, as previously described.^20^,^21^ Ten of the 24 subjects additionally underwent DST imaging as part of the original protocol, which were used for method development (“DST-CAD”).
  2. The remaining 23 subjects in the comparison with CMR were diagnosed with asymptomatic moderate-severe or severe mitral- or aortic regurgitation and were consecutively included into an on-going prospective outcome study. Subjects with regurgitation (“Regurge” in Table 1 and Fig. 3) were scanned with ^15^O-water PET/CT at rest and stand-alone CMR on the same-day.
- The Echo-comparison was done in 36 subjects from two different previous protocols, in which PET-based WT measurements were a pre-specified aim.
  1. One protocol explored the usefulness of PET in cardiac amyloidosis^22^; 11 subjects underwent ^15^O-water PET at rest (CA, *n* = 5; healthy volunteers, *n* = 5; HCM, *n* = 1) and echocardiography on the same-day (“Amyloid+HV” in Table 1 and Fig. 3).
  2. The second protocol included 25 subjects with high-risk hypertrophic cardiomyopathy (“HCM” in Table 1 and Fig. 3), all treated with implantable cardioverter defibrillators.^23^ HCM subjects underwent ^15^O-water PET both at rest and with adenosine-induced stress in one session. A planned echocardiography was performed within a week of PET.

### PET Imaging

A GE Discovery DST PET/CT scanner was used for initial method development (66 scans from 66 subjects, see Fig. 3). A GE Signa PET/MRI scanner was used in the CAD cohort (48 scans in 24 subjects) and a GE Discovery DMI in remaining subjects (83 scans in 59 subjects) with blinded evaluation (all scanners GE Healthcare, Waukesha, WI).

DST, DMI and Signa scanners feature 15, 20 or 25 cm axial field of view, respectively. A scan was performed during 6 minutes starting with a rapid standardized bolus infusion of 400 MBq ^15^O-water (a volume of 3 to 5 mL infused with an autoinjector at 1 mL/s, directly followed by 35 mL saline at 2 mL/s). Data were stored in list mode and reconstructed using a standardized clinical protocol (time frames of 1 × 10, 8 × 5, 4 × 10, 2 × 15, 3 × 20, 2 × 30, 2 × 60 s) into a 128 × 128 matrix with a transaxial field of view of 50 cm (DST and DMI) or 53.4 cm (Signa) using ordered subsets expectation maximization (DST: 2 iterations, 28 subsets (2i28s), 5 mm post-filter; DMI: 3i16s, 5 mm post-filter; Signa: 3i28s, 6 mm post-filter), according to manufacturer’s recommendations. Time-of-flight and point-spread-function recovery were used with DMI and Signa, but were not available with DST. Scanning was repeated with adenosine-induced vasodilation in subjects from the CAD (*n* = 24) and HCM (*n* = 24) cohorts with 12 minutes between ^15^O-water injections. All available scans were used “as is” to mimic a clinical scenario.

### PET-Based Evaluation of LV Function and Geometry

PET scans were analyzed using aQuant (Medtrace Pharma A/S, Lyngby, Denmark).^14^,^18^ LVM and WT were calculated using contouring of parametric PTF images in the short-axis view with modifications to a previously published semi-automatic approach.^19^ Briefly, the LV was rotated to short-axis view and, for each short-axis slice included by the user, the outer and inner contours were defined by identifying points at a predefined fraction at 67% of the maximum value along profiles with 10-degree increments projected from the center of the cavity. LVM was measured as the sum of volumes of voxels fully enclosed by the inner and outer contours, multiplied with 1.05 to account for the specific gravity of soft tissue. WT was measured at the mid-septal level as the average of 5 profiles close to the line connecting the centers of gravity of the LV and RV.

Finally, manual corrections could be performed by the user to correct, for instance, for spillover from abdominal activity, but these were, importantly, not performed on the mid-septal wall from which WT was obtained.

Inter-observer agreement of PET-based LVM and WT measurements was studied in the entire cohort using DMI and Signa scanners. In this analysis the second observer used the full automation and only corrected for poor basal plane definition, if needed. A test-retest analysis was performed by one observer by comparing LVM from a rest scan to LVM from a stress scan in the CAD and HCM cohorts. PET observers were blinded to CMR and echocardiography.

### CMR

Two different MR scanners were used: a 3 Tesla Signa PET/MR (GE Healthcare, Waukesha, WI), simultaneous with PET acquisition, in the CAD cohort, and a 3 Tesla Philips Achevia (Philips Healthcare, Best, The Netherlands) with a standard protocol 1 to 2 hours before PET in the Regurge cohort. LVM was measured in the resting state. Short- and long-axis cine images were acquired using a steady-state free precession pulse sequence. All CMR data were analyzed by one observer, blinded to PET data, using a commercially available CMR software (Philips Viewforum, Best, The Netherlands). LV volumes and mass were determined by a semi-automated segmentation approach of the short-axis stack images using long-axis images to define the most basal slice. End-diastolic endocardial and epicardial contours were propagated with manual re-adjustments performed as required. Papillary muscles and adnexal tissue were not included in LVM. Maximal mid-septal WT was measured from an end-diastolic 4-chamber cine view.

### 2D-echocardiography

Transthoracic 2D examinations used a GE Health Care Vivid 9 (Horton, Norway) echo machine. In the Amyloid-HV cohort septal wall thickness was measured in parasternal long-axis^5^ by one observer on the day of PET scanning, blinded to PET data. In the HCM-cohort the largest wall thickness in the mid-ventricular septum was measured in a short-axis view by one observer within 1 week of PET, blinded to PET data.

### Statistics

Data were evaluated for normal distributions using Shapiro–Wilk tests and Q–Q plots, and presented as mean ± standard deviation (SD) unless otherwise stated. Linear regression analysis was used to calculate correlations and Bland–Altman plots to assess agreement. Post-hoc residual analysis was performed with multivariate approaches and visual inspections. Brown-Forsythe test was used to evaluate differences in variance between groups.

Diagnostic accuracy of ^15^O-water PET in identifying hypertrophy was assessed using institutional normal values for CMR and 2DE as standard of truth. For LVM, CMR hypertrophy was defined as indexed LVM (g/m^2^) > 81 for women and > 85 for men. For both CMR and 2DE WT < 12 mm was used to indicate a normal wall thickness, and WT > 15 mm indicated severely increased wall thickness. Receiver Operating Characteristics (ROC) curve analysis was used to derive an area-under-the-curve (AUC), and define best cut-off values. Contingency tables were used to calculate sensitivity, specificity and accuracy. Agreement of inter-observer and test-retest results was determined using intraclass correlations (ICC) and a repeatability coefficient was calculated (1.96 × SD of differences). A two-sided *P* value < 0.05 was considered significant. Statistical calculations were performed in GraphPad Prism 9 (Graphpad Software Inc, CA). JMP 14 (SAS Institute, San Diego CA) was used for multivariate analyses. Matlab 2020a was used to calculate ICC.

## Results

### Method Development Using DST PET/CT

Results with linear regression and Bland–Altman plots are shown in Supplementary Figure S1. In summary, initial studies in the Regurge cohort showed that a 67% cut-off for PTF segmentation resulted in significant overestimation of LVM and WT with this older scanner. Increasing the cut-off to 80% resulted in high correlations for LVM versus CMR of *r* = 0.82 (*P* < 0.0001) in the Regurge cohort and *r* = 0.91 (*P* = 0.0003) in the 10 subjects from the DST-CAD cohort, but with a significant and negative proportional bias in the pooled cohort (*r* = − 0.51, *P* < 0.001). DST-derived WT correlations versus CMR were *r* = 0.55 (*P* = 0.0007) in Regurge subjects and *r* = 0.73 (*P* = 0.02) in DST-CAD subjects with a pooled systematic bias of 2.3±2.0 mm. Notably, a range of cut-offs from 67% to 85% produced identical results for LVM and WT in the DST-CAD cohort. DST-WT versus 2DE in the Amyloid-HV cohort showed a correlation of *r* = 0.78 (*P* < 0.0001, *n* = 23) with no significant bias. Based on these slightly conflicting results a decision was made to proceed with a 67% cutoff for the blinded analyses with the newer scanners.

### Blinded Comparison of PET to CMR

**Figure 4:**
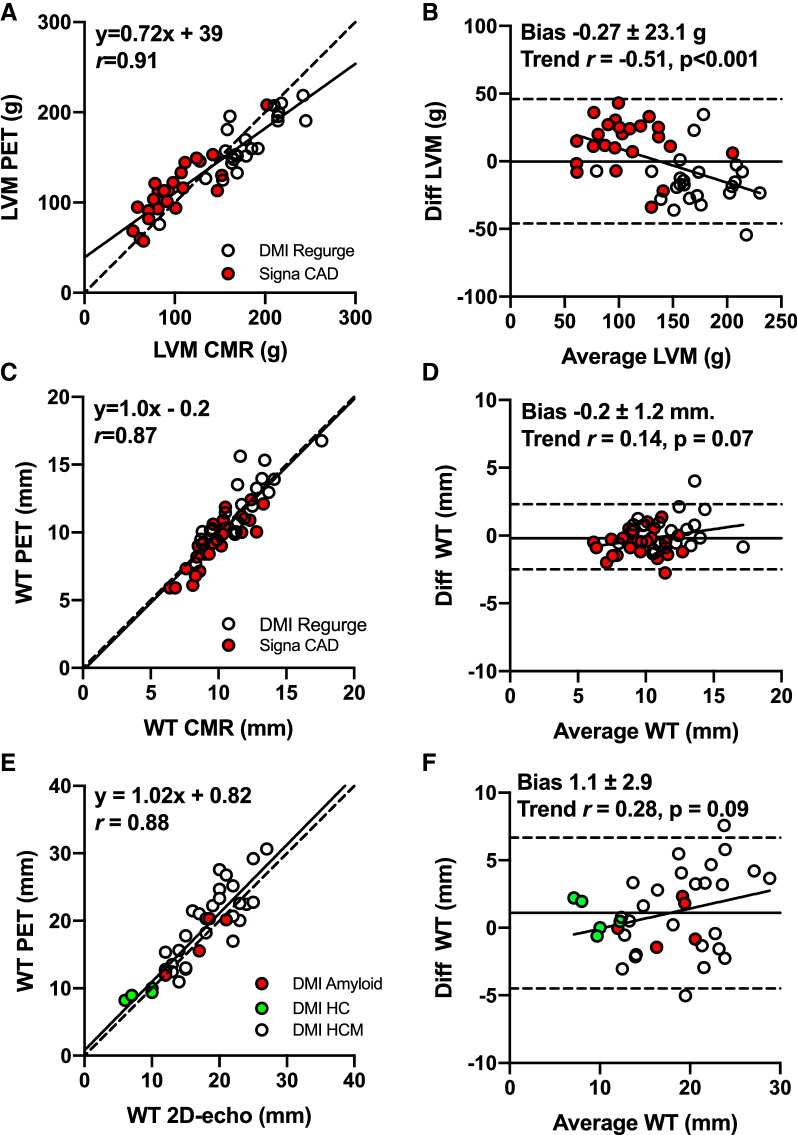
LV mass (LVM) and septal wall thickness (WT) for ^15^O-water PET versus cardiac magnetic resonance (CMR, **A**-**D**)) and WT for ^15^O-water PET versus 2D-echocardiography (2D-echo, **E**-**F**). **A**, **C**, **E** Linear regression analysis, dotted line is line of unity. **B**, **D**, **F** Bland-Altman plots, stippled horizontal lines show limits of agreement and line of correlation show proportional bias. Signa: GE Signa PET/MR). DMI: GE Discovery MI PET/CT. Diff: PET-CMR. Average: (PET+CMR)/2

Results of the analysis of LVM and WT from DMI and Signa scanners are shown in Fig. 4. A high correlation of LVM towards CMR was found (*r* = 0.91, *P* < 0.0001, Fig. 4A) and was equally good with both PET systems (both *r* = 0.86, *P* < 0.0001). Systematic bias in the pooled data set was insignificant (Figure 4B), but LVM was overestimated by PET in the CAD cohort (paired t-test: 14 g (95% CI 6 to 21), *P* = 0.001) and underestimated in the Regurge cohort (− 23 g (95% CI − 11 to − 35), *P* = 0.0005), resulting in a significant proportional bias in the pooled data (*r* = − 0.51, *P* < 0.001 for trend). LVM residuals were analyzed with a stepwise multiple regression approach and were largely explained by variation in CMR-derived end-diastolic volumes (improvement of *r* from 0.91 to 0.95, *P* < 0.0001).

PET-WT derived from Signa and DMI PET data had similar and high correlations (both *r* > 0.85) towards CMR with no significant deviations from the line of unity (Fig. 4C) and no significant bias (Fig. 4D).

### Blinded Comparison of PET to Echocardiography

PET-WT correlated well with 2DE (*r* = 0.88, *P* < 0.0001, Fig. 4E) with no significant systematic bias (1.1 ± 2.9 mm, *P* = 0.1) or proportional bias (*r* = 0.28, *P* = 0.08,), shown in Fig. 4F. Differences were smaller in subjects with evenly distributed wall thickness (“Amyloid +HV” cohort) than in “HCM”, where the majority had heterogenous distribution of hypertrophy (Brown-Forsythe test *P* = 0.003).

### Diagnosing LV Hypertrophy

Results with 95% confidence intervals for ROC curve and contingency table analyses are detailed in Table 2. Increased LVM was diagnosed by CMR in 10 of 47 subjects and the AUC for indexed PET-LVM was 0.97 (*P* < 0.0001) with 1 false positive (FP) and 1 false negative (FN). The best cut-off was 85.1 g/m^2^, resembling the upper normal limit for CMR (81 g/m^2^ for females and 85 g/m^2^ for males), and resulted in a high accuracy of 0.96. WT larger than 12 mm was diagnosed by CMR in 11 of 47; the AUC of PET-WT was 0.91 with 3 FP and 2 FN at best cut-off 11.9 mm, resulting in an accuracy of 0.89 (*P* < 0.0001).

**Table 2:** ROC curve analysis of non-ECG-gated ^15^O-water PET for observer-blinded detection of left ventricular hypertrophy

Parameter	*N* true positive /total	AUC	Best PET cutoff	Sensitivity	Specificity	Accuracy
Increased LV mass (CMR)	10/47	0.97 (0.92–1.00)	> 85.1 g/m^2^	0.90 (0.60–0.99)	0.97 (0.86–1.00)	0.96 (0.86–0.99)
Increased wall thickness > 12 mm (CMR)	11/47	0.91 (0.82–1.00)	> 11.9 mm	0.82 (0.52–0.97)	0.92 (0.78–0.97)	0.89 (0.77–0.95)
Increased wall thickness > 12 mm (CMR+2DE)	39/83	0.95 (0.79–0.99)	> 12.1 mm	0.90 (0.76–0.96)	0.89 (0.76–0.95)	0.89 (0.81–0.94)
Severely increased wall thickness > 15 mm (CMR+2DE)	22/83	0.997 (0.99–1.00)	> 15.5 mm	1.00 (0.85–1.00)	0.95 (0.87–0.99)	0.96 (0.90–0.99)

Numbers in parentheses are 95% confidence intervals

*AUC*, area under curve

PET-based WT was further tested on two levels in the pooled cohort from CMR and 2DE: 12 mm, defining an upper normal limit, and 15 mm, defining severely increased wall thickness. CMR and 2DE combined detected WT > 12 mm in 39 of 83 subjects and WT >15 mm in 22 of 83. AUC at the 12 mm level was 0.95 (*P* < 0.0001) with 5 FP and 4 FN; best cut-off was 12.1 mm and resulted in an accuracy of 0.89. AUC at the 15 mm level was near-perfect at 0.997 (*P* < 0.0001) with 3 FP and no FN; best cut-off was 15.5 mm, resulting in an accuracy of 0.96.

### Reproducibility

**Figure 5:**
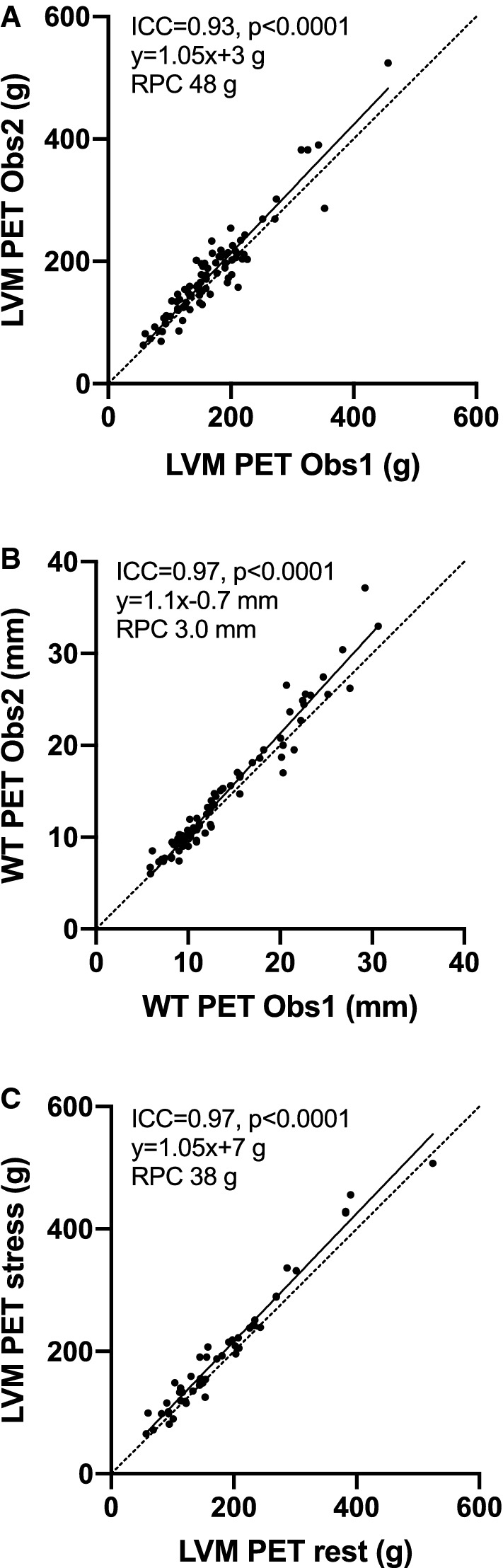
Reproducibility for ^15^O-water PET with standardized LV wall segmentation. **A** Inter-observer variation of LV mass (LVM). **B** Inter-observer variation of septal wall thickness (WT). **C** Test–retest repeatability of LVM at rest and during adenosine-induced vasodilation (stress). Obs1, Obs2: PET observers. ICC: intra-class correlation. RPC: repeatability coefficient

Results of reproducibility studies are shown in Table 3 and Fig. 5. Manual corrections generally only changed the results by a few percentages, compared to full automation. More extensive corrections were needed in a few cardiomyopathic subjects with generalized fibrosis. Agreement was high both in inter-observer reproducibility of LVM and WT (Fig. 5A-B) and rest-stress repeatability of LVM (Fig. 5C) with excellent ICC values (lower boundaries of 95% CI were ≥ 0.87 in all, *P* < 0.0001) and small repeatability coefficients (see Table 3). PET image analysis was performed on standard laptops; once the original images were loaded an automated analysis took approximately 2 min to finalize and save the report, while manual corrections added 1 to 3 minutes to processing time.

**Table 3:** Inter-observer and test-retest analyses

	*N*	ICC (95% CI)	Bias	RPC (%)
Inter-observer reproducibility				
LV mass	83	0.93 (0.87–0.96)	12±25 g	48 g (26%)
Septal wall thickness	83	0.97 (0.95–0.98)	0.7±1.6 mm	3.0 mm (18%)
Test-retest repeatability (rest/stress with one observer)				
LV mass	48	0.97 (0.87–0.99)	15±20 g	38 g (24%)

*ICC*, intra-class correlation; *RPC*, repeatability coefficient

## Discussion

This study introduces the ability to diagnose LV hypertrophy as spin-off marker from a ^15^O-water PET scan, performed with the primary purpose of quantifying myocardial blood flow. The workflow is fast with user input mainly required for quality control and show highly reproducible results. As it makes use of static parametric images instead of ECG-gated late uptake images, it is in theory suitable for most PET perfusion tracers whether these tracers are retained (e.g., ^11^C-acetate) in the LV wall or not (e.g., ^15^O-water).

We tested and validated the method in subjects with various cardiac geometries, ranging from normal to advanced hypertrophy with both eccentric and concentric remodeling patterns, and found substantial agreement compared to CMR and echocardiography. As expected, contemporary scanners showed higher agreement than an older system.

Cardiac PET is increasingly used for evaluation of myocardial ischemia, but is typically requested late in the diagnostic process when a potential diagnosis of LV hypertrophy should be known from a routinely performed echocardiography. Recent ESC guidelines recommend echocardiography or, if echocardiography is indeterminate, CMR at baseline for detection of concomitant structural heart disease before applying cardiac imaging for CAD evaluation.^24^ However, in clinical practice we have encountered several scenarios where the evaluation of LV hypertrophy directly from a cardiac perfusion PET scan is of potential clinical relevance:

- Reassessment of LV hypertrophy in subjects with poor acoustic windows at previous echocardiography; of particular relevance in patients with contraindications for CMR.
- Evaluation of LV hypertrophy as a cause of reduced stress perfusion when PET is performed before echocardiography or when PET is performed long after echocardiography.
- Monitoring in subjects when therapy aims at reversing both poor myocardial blood flow and structural remodeling, for example in the prevalent situation of non-obstructive CAD with low perfusion reserve and hypertrophied hearts in heart failure with preserved ejection fraction or in the increasing number of subjects with significant CAD and hypertrophy treated medically.

The use of PTF instead of MBF for myocardial delineation effectively uncouples partial volume effects from perfusion,^17^ making the approach applicable even in the presence of severe perfusion abnormalities during stress (Fig. 2). PTF is regionally lowered in myocardial scarring, but rarely to the extent that the scarred regions cannot be visualized.^18^ PTF is also affected by PET-CT misalignment at reconstruction,^25^ which was not corrected for in the current study cohorts. Errors due to misalignment might explain some of the variation in the data. Regardless, PET-LVM correlated well with CMR for all three PET systems in the study and can potentially be of use even with older scanners. PET-WT was highly accurate for the newer scanners, compared to CMR, but performed poorer in the normal WT range with the older system.

We used a pre-specified and uniform segmentation threshold at 67% of maximum PTF along radial projections in all subjects, which resulted in high septal PET-WT agreement and diagnostic accuracy for both DMI and Signa PET scanners in both eccentric and concentric hypertrophy. The agreement was slightly lower in HCM patients with heterogenous wall thickness, potentially because PET automatically measured WT in a septal geometrical midpoint while the 2DE observer measured the largest WT detected anywhere in the midventricular septum.

There was a negative proportional trend in LVM (Fig. 4B), which was seen with all three scanners and slightly more pronounced with the DST scanner. This trend was largely explained by variation in LV cavity size, suggesting that PTF images overestimate wall thickness in normal sized hearts and underestimates in eccentric hypertrophy. However, as septal PET-WT was equal to CMR WT in both the CAD and the Regurge cohorts with the newer scanners, the under- or overestimation either occurred in non-septal regions or could be caused by suboptimal sectioning at the valve plane, which is the region of an ECG-averaged heart image most affected by motion. CMR ruled out extensive subendocardial fibrosis in all subjects from the Regurge cohort, which could otherwise explain the finding in part. Additionally, CMR LVM measurements excluded trabecular and papillary muscle tissue; exact separation of tissue components is currently beyond the capacity of non-gated PET images, even with solid-state detectors, and most likely degraded LVM agreement. Interestingly, a similar high correlation and a negative proportional trend was seen when ECG-gated 82Rb PET was compared to CMR in a recent study by Malahfji et al.,^8^ suggesting that 82Rb and ^15^O-water perform similarly for LVM assessments even though 82Rb uses ECG-gated uptake images and 15O-water static parametric images. On the other hand, in the only previous study using parametric images to calculate LVM, based on non-gated ^11^C-acetate PET in subjects with aortic stenosis and in healthy volunteers, a small positive proportional bias was found. The apparent variation could be due to differences in PET devices and the different tracers, but CMR values are also susceptible to fluctuation between centers.^26^

The ROC analysis showed a best PET LVM diagnostic cutoff at 85 g/m^2^, which was similar to the cutoff used clinically with CMR in our institution. A uniform 67% segmentation with images from newer scanners thus results in appropriate cut-offs for abnormal LVM and WT, but at the expense of overestimation of LVM in small hearts and underestimation in extreme hypertrophy. With images from the older DST PET/CT used for method development a best cutoff at 80% was found to work moderately well for LVM estimates in dilated hearts, but was associated with increased proportional bias, and had poorer accuracy for detection of mildly increased septal hypertrophy. Considering that the 67% cutoff performed very well when the same method was previously applied to non-gated ^11^C-acetate images on a mid-range scanner with point-spread-function and time-of-flight reconstructions^19^ we assume that the 67% cut-off will produce acceptable results on the vast majority of currently installed PET/CT systems, but confirmatory studies are needed.

The measurement precision of the new method was assessed in two ways: a standard inter-observer analysis and a combined intra-observer/rest-stress analysis. Inter-observer agreement was excellent (ICC = 0.93 for LVM and ICC = 0.97 for WT). Similarly, rest-stress LVM agreement was excellent at ICC = 0.97 with unexpectedly small variation even among the HCM subjects with severe regional perfusion abnormalities. This finding supports the notion that myocardial morphology and perfusion can be separated by ^15^O-water PET, which, as shown in Fig. 2, could be important for visualizing the presence and extent of subendocardial ischemia. LVM reproducibility of ^15^O-water PET was in line with recent reports using 82Rb.^9^,^10^

### Limitations

This was a single center and first proof-of-principle study in different types of cardiac remodeling and with different scanners. The findings are encouraging but need prospective external validation on more scanner types for more wide-spread clinical use. Normal LVM values differ with sex, age, and ethnicity^27^ but would require much larger and diverse material for characterization. The accuracy of PET LVM was not determined in concentric hypertrophy, as LVM measurements by 2DE in HCM are not appropriate and CMR was not performed. 2DE was also used to assess the accuracy of WT in healthy controls and primary cardiomyopathies, which is not an ideal gold standard, but is the first-choice imaging modality in relevant guidelines.

## Conclusion

Parametric PTF images derived from a standard clinical ^15^O-water myocardial perfusion PET scan on newer scanners provide accurate and precise estimates of left ventricular mass and septal wall thickness. Accuracy was reduced on an early-generation PET/CT.

## New Knowledge Gained

Left ventricular hypertrophy in terms of LVM and mid-septal WT can be assessed with high accuracy from a single ^15^O-water PET scan.

## Supplementary Information

Below is the link to the electronic supplementary material.

Supplementary material 1 (DOC 145 kb)

Supplementary material 2 (PPTX 20606 kb)

## Abbreviations

MBF: Myocardial blood flow
PTF: Perfusable tissue fraction
LV: Left ventricle
LVM: Left ventricular mass
WT: Septal wall thickness
PET: Positron emission tomography
CMR: Cardiac magnetic resonance imaging
2DE: 2D echocardiography
CAD: Coronary artery disease
ROC: Receiver operating characteristics
AUC: Area under the curve
ICC: Intraclass correlation
