# Supplementary material for: Diagnosis of left ventricular hypertrophy using non-ECG-gated 15O-water PET
Source: J Nucl Cardiol. 2021 Jul 20;29(5):2361–73. doi: 10.1007/s12350-021-02734-3 (PMC9553817; doi:10.1007/s12350-021-02734-3)
Supplement: Supplementary file 1 — Supplementary material 1 (DOC 145 kb) [file 12350_2021_2734_MOESM1_ESM.doc]

Supplementary material - Diagnosis of left ventricular hypertrophy using non-ECG-gated ^15^O-water PET

**Suppl. Figure S1.** Linear regression and Bland-Altman analyses of PET-data from GE Discovery ST (DST). These data sets were used for experimental and learning purposes. The results in these plots were obtained by one observer using the final software version, blinded to results from CMR and 2D-echocardiography. *A, B*: Comparison of LV mass (LVM) from DST versus CMR. *C, D*: Comparison of septal wall thickness versus CMR. *E, F*: Comparison of WT versus 2D-echocardiography. Stippled lines left column: line of unity, right column: limits of agreement. Regurge: advanced LV regurgitation (n=33). CAD: known or suspected coronary artery disease (n=10). Amyloid: known cardiac amyloidosis (n=11), HCM: known hypertrophic cardiomyopathy (n=6). HC: healthy controls (n=4).
