# Supplementary material for: Diagnosis of left ventricular hypertrophy using non-ECG-gated 15O-water PET
Source: J Nucl Cardiol. 2021 Jul 20;29(5):2361–73. doi: 10.1007/s12350-021-02734-3 (PMC9553817; doi:10.1007/s12350-021-02734-3)
Supplement: Supplementary file 2 — Supplementary material 2 (PPTX 20606 kb) [file 12350_2021_2734_MOESM2_ESM.pptx]

## Slide 1
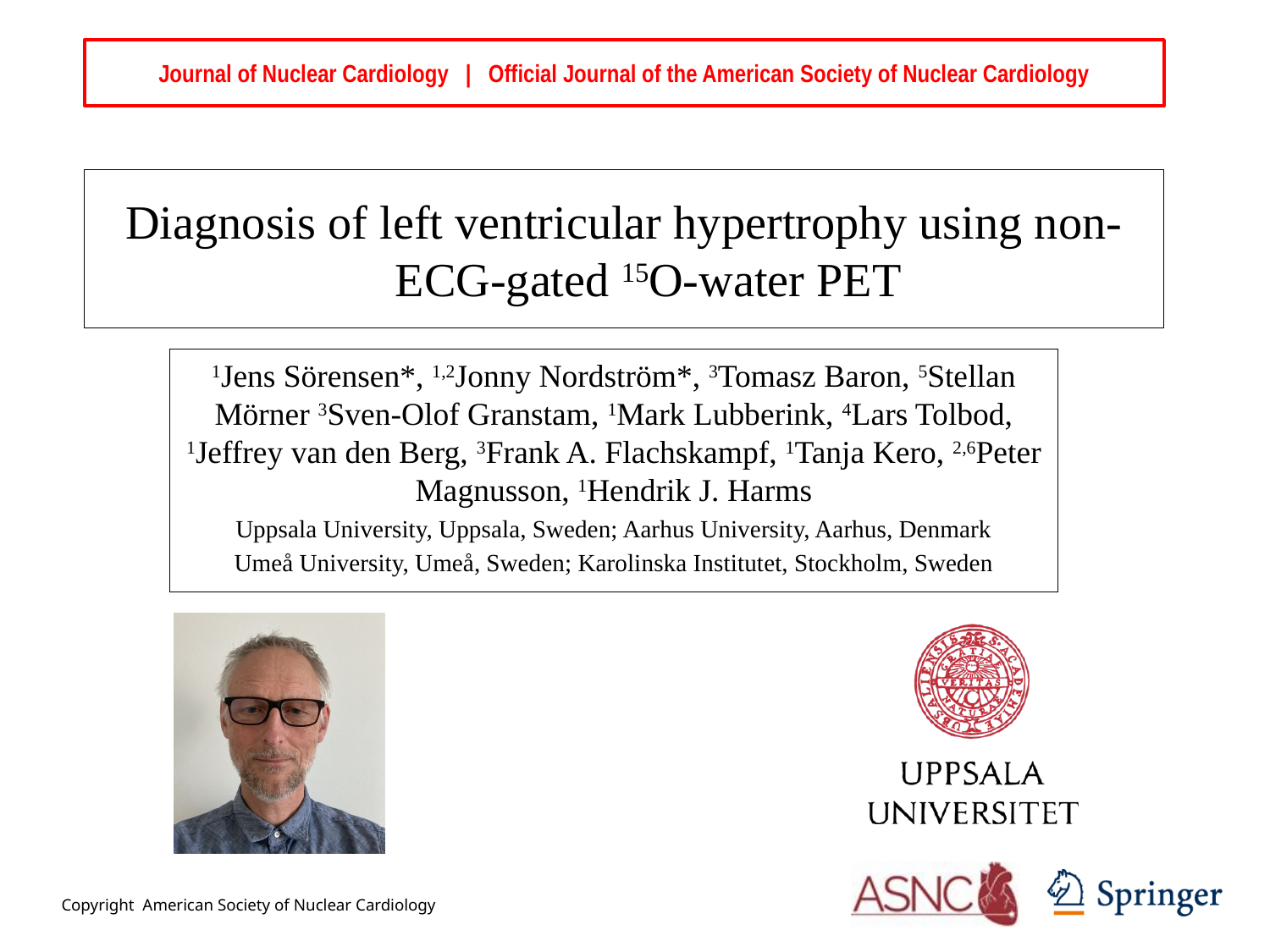

Journal of Nuclear Cardiology | Official Journal of the American Society of Nuclear Cardiology
# Diagnosis of left ventricular hypertrophy using non-ECG-gated 15O-water PET
1Jens Sörensen*, 1,2Jonny Nordström*, 3Tomasz Baron, 5Stellan Mörner 3Sven-Olof Granstam, 1Mark Lubberink, 4Lars Tolbod, 1Jeffrey van den Berg, 3Frank A. Flachskampf, 1Tanja Kero, 2,6Peter Magnusson, 1Hendrik J. Harms
Uppsala University, Uppsala, Sweden; Aarhus University, Aarhus, Denmark
Umeå University, Umeå, Sweden; Karolinska Institutet, Stockholm, Sweden
Copyright American Society of Nuclear Cardiology

## Slide 2
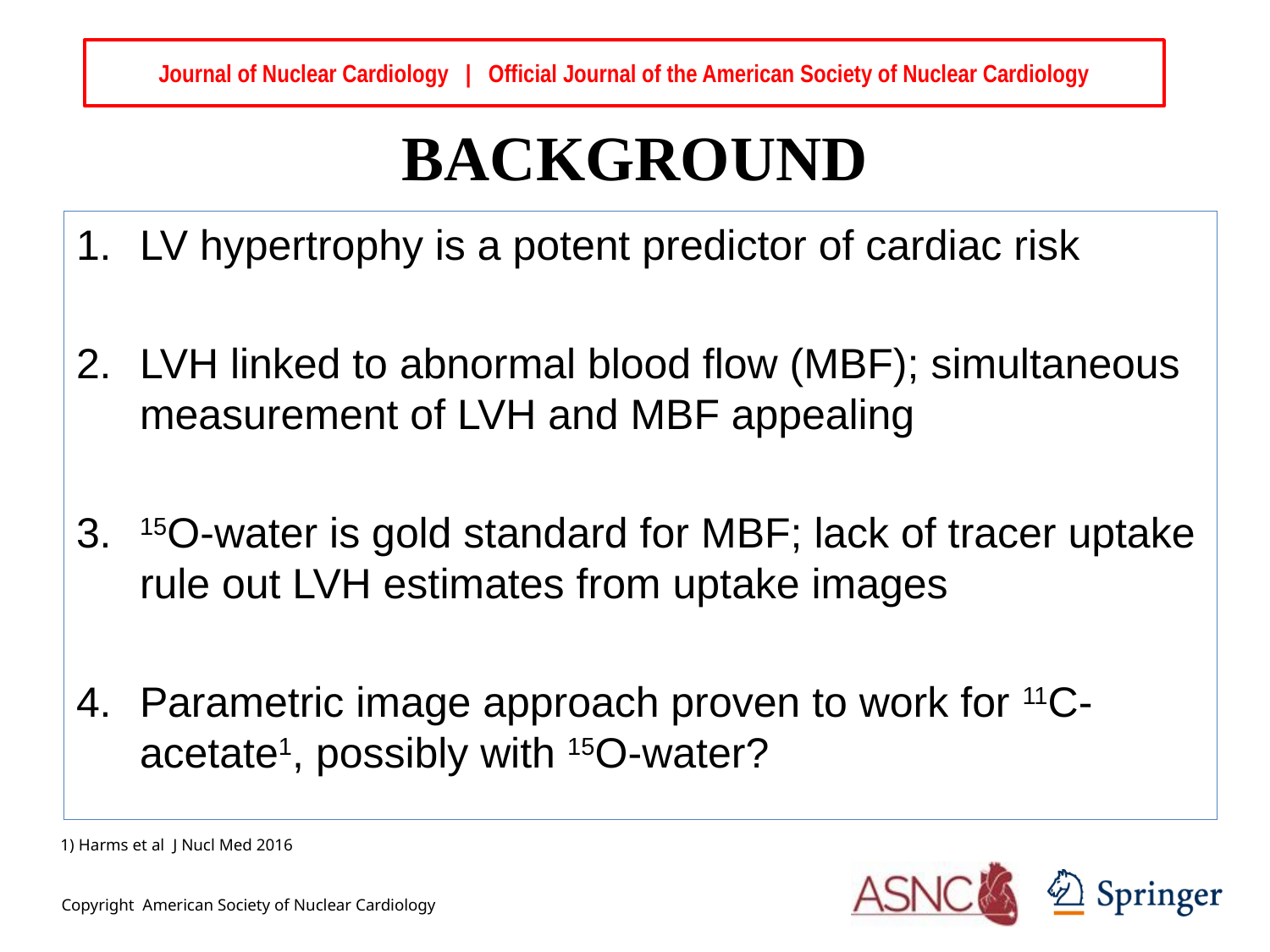

Journal of Nuclear Cardiology | Official Journal of the American Society of Nuclear Cardiology
# BACKGROUND
LV hypertrophy is a potent predictor of cardiac risk
LVH linked to abnormal blood flow (MBF); simultaneous measurement of LVH and MBF appealing
15O-water is gold standard for MBF; lack of tracer uptake rule out LVH estimates from uptake images
Parametric image approach proven to work for 11C-acetate1, possibly with 15O-water?
1) Harms et al J Nucl Med 2016
Copyright American Society of Nuclear Cardiology

## Slide 3
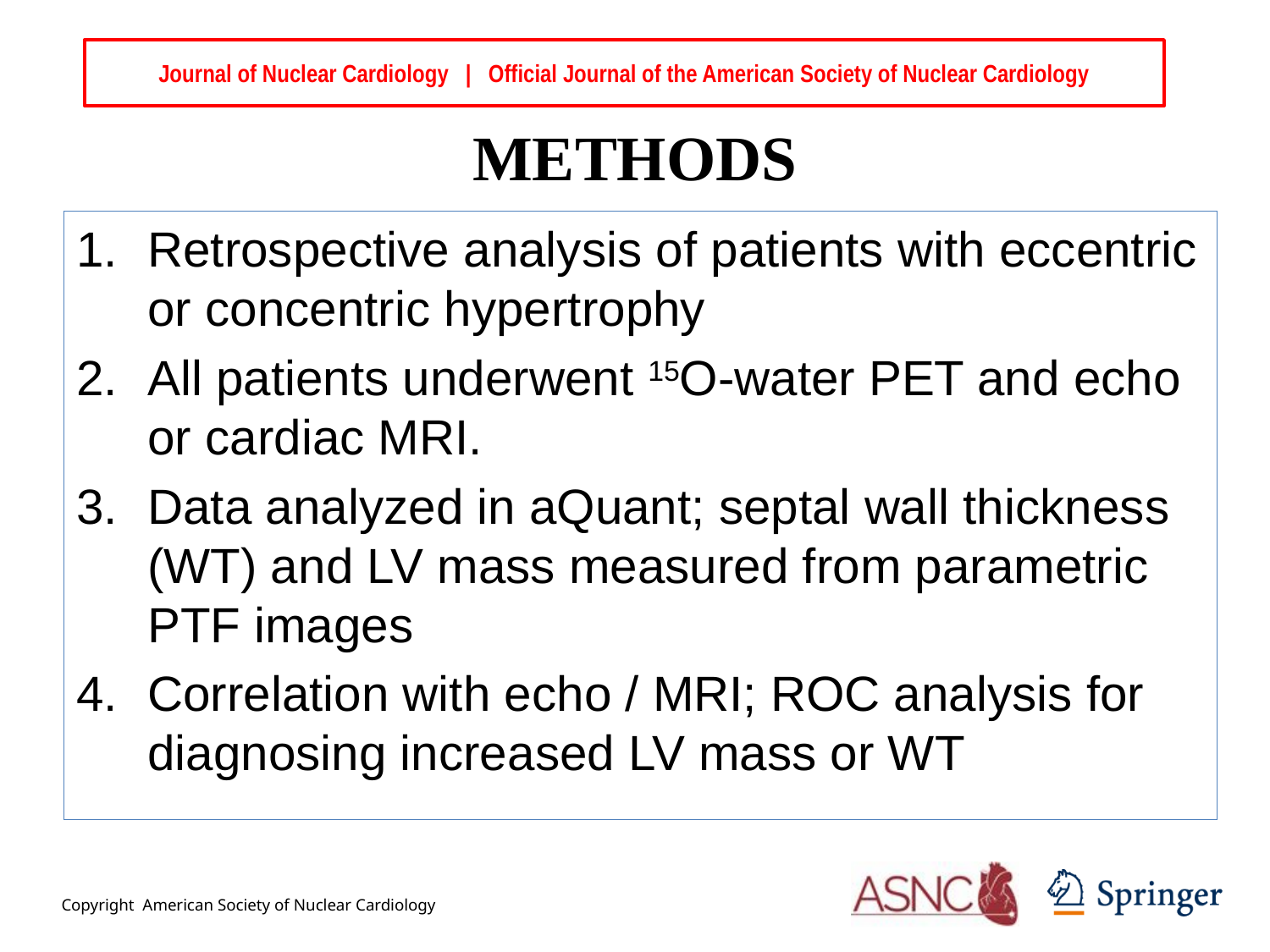

Journal of Nuclear Cardiology | Official Journal of the American Society of Nuclear Cardiology
# METHODS
Retrospective analysis of patients with eccentric or concentric hypertrophy
All patients underwent 15O-water PET and echo or cardiac MRI.
Data analyzed in aQuant; septal wall thickness (WT) and LV mass measured from parametric PTF images
Correlation with echo / MRI; ROC analysis for diagnosing increased LV mass or WT
Copyright American Society of Nuclear Cardiology

## Slide 4
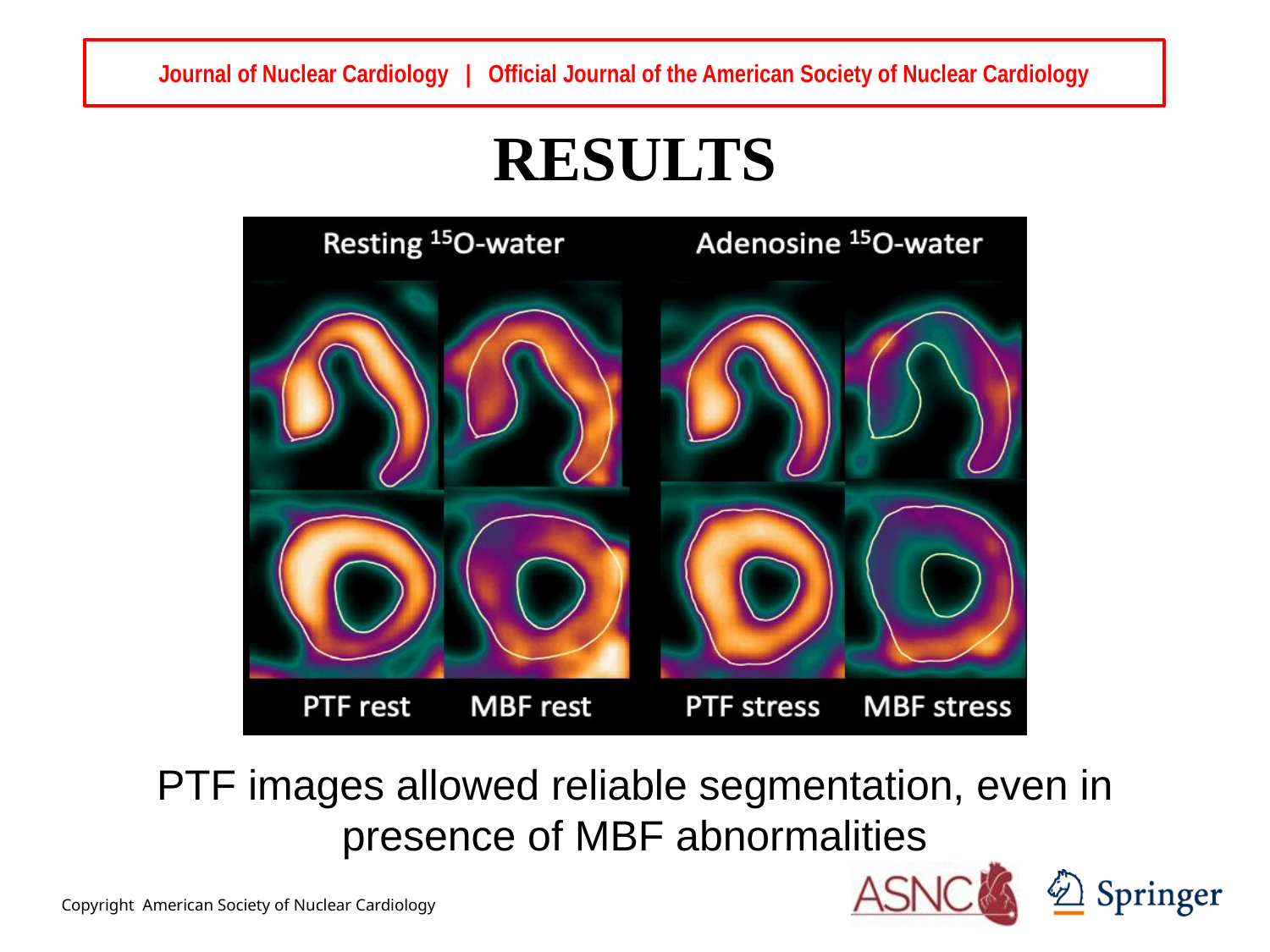

Journal of Nuclear Cardiology | Official Journal of the American Society of Nuclear Cardiology
# RESULTS
PTF images allowed reliable segmentation, even in presence of MBF abnormalities
Copyright American Society of Nuclear Cardiology

## Slide 5
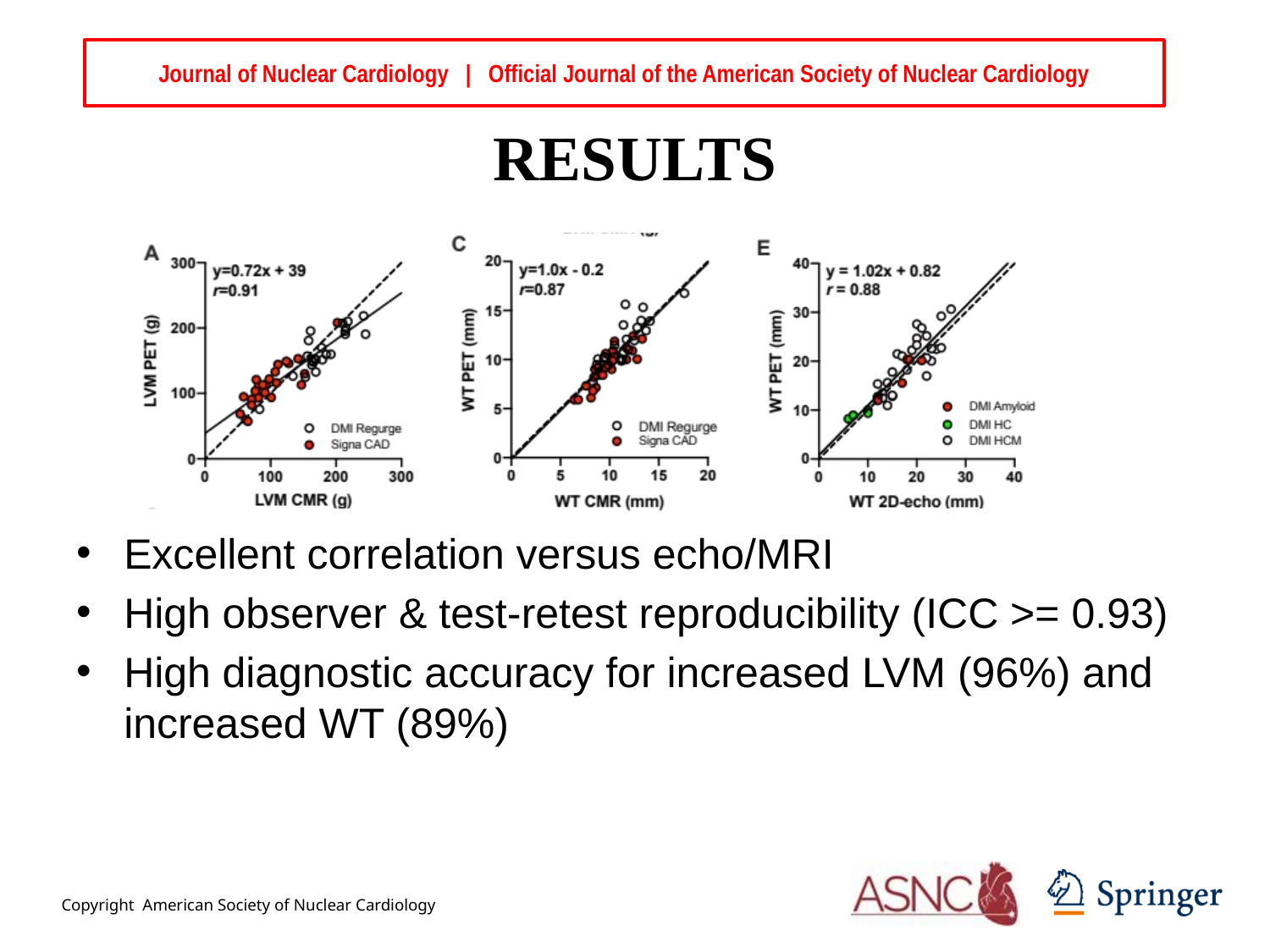

Journal of Nuclear Cardiology | Official Journal of the American Society of Nuclear Cardiology
# RESULTS
Excellent correlation versus echo/MRI
High observer & test-retest reproducibility (ICC >= 0.93)
High diagnostic accuracy for increased LVM (96%) and increased WT (89%)
Copyright American Society of Nuclear Cardiology

## Slide 6
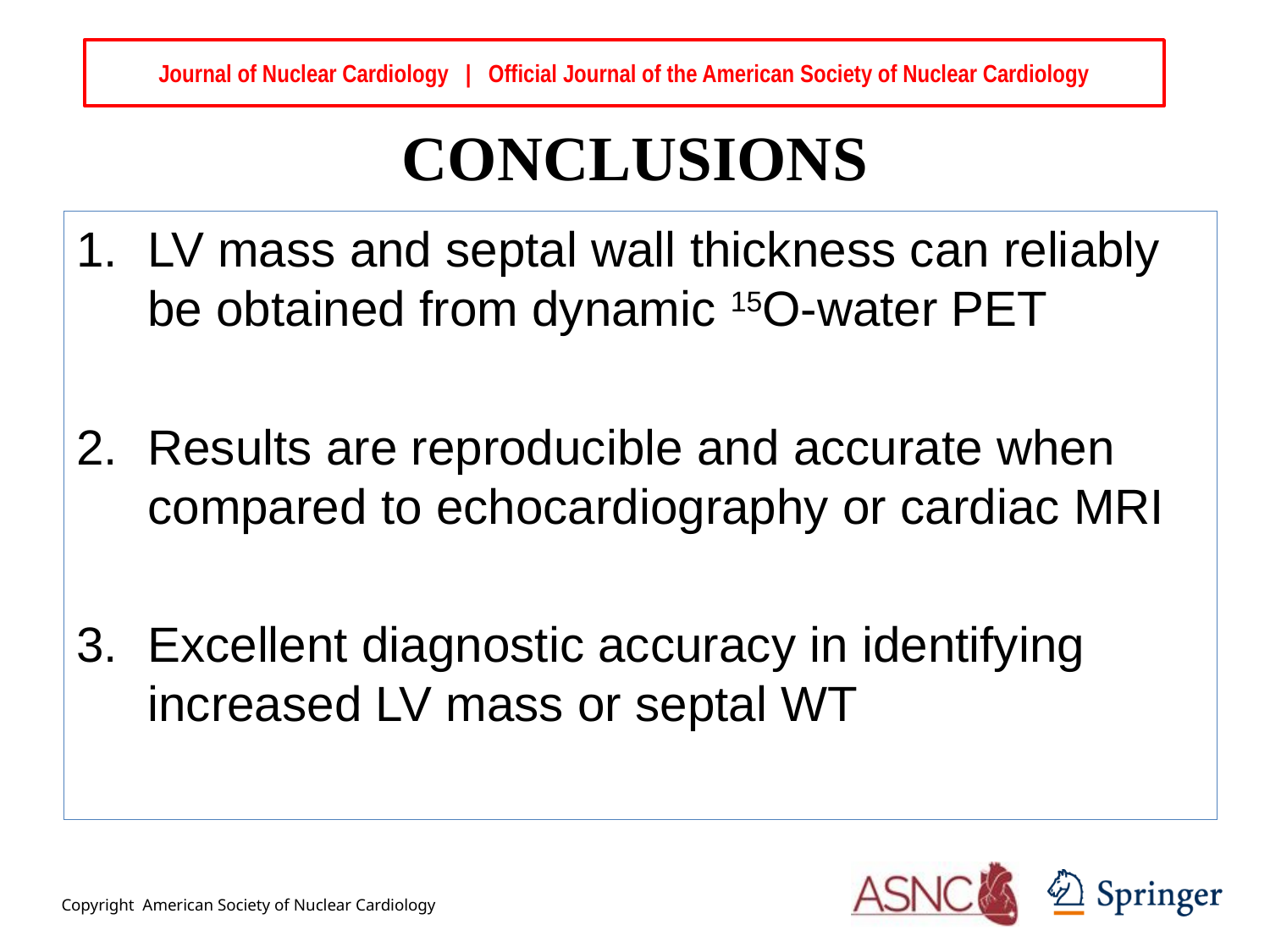

Journal of Nuclear Cardiology | Official Journal of the American Society of Nuclear Cardiology
# CONCLUSIONS
LV mass and septal wall thickness can reliably be obtained from dynamic 15O-water PET
Results are reproducible and accurate when compared to echocardiography or cardiac MRI
Excellent diagnostic accuracy in identifying increased LV mass or septal WT
Copyright American Society of Nuclear Cardiology
